# The Influence of Autohydrolysis Temperature and the Addition of 2 wt% of Expired Paracetamol on the Thermal Behavior and Composition of Pyrolysis Products After Hydrothermal Treatment of Sunflower Stems (SSs) and Sunflower Inflorescences (SIs)

**DOI:** 10.3390/molecules31081236

**Published:** 2026-04-09

**Authors:** Andrzej Strojwas, Valentina Zubkova, Joanna Masternak, Ilona Stabrawa

**Affiliations:** 1 Institute of Chemistry, Jan Kochanowski University in Kielce, Uniwersytecka Str. 7, 25-406 Kielce, Poland; andrzej.strojwas@ujk.edu.pl (A.S.); joanna.masternak@ujk.edu.pl (J.M.); 2 Institute of Physics, Jan Kochanowski University in Kielce, Uniwersytecka Str. 7, 25-406 Kielce, Poland; ilona.stabrawa@ujk.edu.pl

**Keywords:** autohydrolysis, hydrochar pyrolysis, expired paracetamol

## Abstract

The influence of the autohydrolysis temperature of sunflower stems (SSs) and sunflower inflorescence (SI) on the changes in the composition of the pyrolysis products of their hydrochars (HCs) was investigated. This research was carried out using a TG/FT-IR analytical device, the semi-quantitative ATR technique, the quantitative XRD technique, and the SEM (EDS) technique. It was found that a rise in autohydrolysis temperature alarmingly increases the contribution of undesirable hydrocarbons in the volatile pyrolysis products of HCs calculated with respect to the emitted CO_2_ and substantially decreases the yield of pyrolyzed solid products. The rise in autohydrolysis temperature not only changes the content of inorganics in HCs but also influences the migration of inorganics in these samples during pyrolysis: intensifies the migration of Mg and Ca and reduces the migration of K. This affects the secondary reaction between the volatile pyrolysis products. The addition of 2 wt% of paracetamol to pyrolyzed HCs inhibits the migration of Mg and Ca and increases the migration of K with volatile products, which positively influences the reduction in undesirable compounds in the composition of emitted volatile products. The addition of paracetamol decreases the yield of pyrolyzed SSHCs by circa 2% and increases the yield of pyrolyzed SIHC_180_ by almost 5%.

## 1. Introduction

The limitations of conventional energy sources are the reason for their replacement by other hydrocarbon sources of plant origin in the transition toward carbon neutrality. Plant biomass, including agricultural lignocellulosic wastes, currently plays a significant role in the development of polygeneration technologies adapted to the circular economy, which thus allows for various types of biofuels and platform chemicals to be obtained [1,2,3]. The use of selective catalysts in pyrolysis processes of biomass allows for controlling the composition of pyrolysis products by changing reaction pathways in the desired direction [4,5]. In addition to basic organic components such as hemicellulose, cellulose, and lignin, agricultural wastes also contain inorganic species [6,7,8,9].

The data in the literature prove that there are some interactions taking place between the organic components of biomass that influence the yield of pyrolysis products [10,11]. The nature and effects of these interactions are presented in various ways by scientists. For example, a range of authors ascertain that the yield of char during pyrolysis can increase as a result of the interaction between cellulose and lignin [10,12,13], and others believe that it decreases [14], whereas the authors of [15,16] suggest that these interactions do not have any effect on the yield of char. The contradictions in the obtained research results draw attention from the viewpoint of the yield of volatile pyrolysis products, which results from the interaction between cellulose and lignin. According to [10,14,17], the yield can increase or decrease [18]. These contradictory results can be found in discussions about the influence of the same inorganics on the course of biomass pyrolysis: potassium and magnesium compounds can increase [19] or decrease [20,21] the yield of volatile products, just as calcium and potassium compounds can increase [9,21] or decrease [19,20] the yield of char.

These interactions can also occur between organic and inorganic biomass components. The inorganic components present in biomass, primarily alkali and alkaline earth metals (AAEMs), can influence the thermal behavior of individual biomass components [22,23] as well as the mechanism of interaction between components in native lignocellulosic biomass [8,24,25,26,27]. According to Guidicianni [13], the complex structure and composition of lignocellulosic biomass and the interactions between organic components, as well as between organic and inorganic components, do not allow for predictions of the final effects of thermal processing of native biomass.

The AAEMs present in the composition of biomass have the ability to undergo migration, emission, and volatilization during its thermal processing. During pyrolysis of biomass, up to 53–76% of alkali metals and 27–40% of alkaline earth metals can be released, while during the gasification of char, 12–34% of alkali metals and 12–16% of alkaline earth metals are released [28]. Online measurements [29] imply that potassium is released to a greater extent during pyrolysis than in the CO_2_ gasification environment at temperatures above 750 °C. Leijenhorst et al. [30] prove that, during pyrolysis of biomass, alkali metals Na and K can be transferred by reactions with volatile substances, while alkaline earth metals (Ca and Mg) and transition metals (Fe, Cu, Ni, Cd, Cr, Co, Mn, and Zn) can remain in solid char. It remains unclear which inorganics, when transferred with volatile pyrolysis products, have a catalytical effect on the secondary reactions between them [31].

Na and K, present in biomass and volatilized during thermal processing, get into bio-oil, cause its instability, and poison the catalysts used in biorefineries [30]. The released AAEMs may have a hazardous effect during pyrolysis, gasification, and combustion due to the formation of sediments on equipment, corrosion of reactors, or the occurrence of molten phases [32,33,34,35,36,37]. In addition, another negative phenomenon occurring during biomass combustion is the formation of particulate matter [38,39]. The research conducted by Turn et al. [40] shows that the main components of emitted particulate matter are C, K, Cl, and S, wherein C atoms may constitute from 50% to 70% of its make-up. According to Yang et al. [41], the emission of particulate matter during the combustion of biomass results from the presence of Na, K, S, Cl, Si, and Al atoms in biomass. The above-mentioned undesirable effects imply that the presence of inorganics in biomass can be a negative factor that exerts influence on its thermal processing.

For that reason, some technological operations aimed at the removal of inorganics from biomass during pretreatment have been implemented. It was stated that pretreatment in the form of washing the biomass with deionized hot liquid water (i.e., hydrothermal treatment called autohydrolysis) removes potassium, amorphous silica, and ash effectively [35,36,37,42] and increases the higher heating value of the obtained hydrochar [43,44,45,46].

After hydrothermal treatment of wheat straw with HCl, Li et al. [47] noticed that the removal of AAEMs from char not only does not change its interaction with volatile substances but also increases the yield of non-condensable gas and reduces the amount of detectable organic compounds in bio-oil. Despite the statement made by X. Tian et al. [48] about the significant influence exerted by leaching with strong acid on the physicochemical structure of biomass, Cen et al. [49] did not observe any significant influence of pre-washing on the changes in the physicochemical properties of corn stems; the researchers only ascertained the removal of some ash and metallic species.

The above-mentioned inconsistencies give grounds for a deeper consideration of the role of intrinsic inorganics removed during autohydrolysis in the interaction processes occurring in biomass at various stages of thermochemical processing. Therefore, it is accepted that lignocellulosic biomass should be subjected to hydrothermal pretreatment in order to refine it by reducing the amount of ash, which will cause an increase in its calorific value [50]. Therefore, the issue connected with hydrothermal processing of lignocellulosic biomass has recently attracted the attention of an increasing number of researchers, as evidenced by the impressive growth in the number of related scientific publications [51]. In addition to autohydrolysis, such operations as hydrothermal liquefaction [52,53], hydrothermal carbonization [54,55,56], hydrothermal gasifications [57,58,59], and others are applied in the thermochemical processing of biomass.

Hydrothermal pretreatment has been classified as a polygeneration technology because its final products can be used to produce biofuels, to obtain platform chemicals, and for other purposes. The aqueous phase in hydrothermal treatment, which contains furans, aldehydes, ketones, organic acids, and phenolic compounds [53,60], is a good raw material for methane fermentation processes after being mixed with hydrochar [61]. However, Wang et al. [62] believe that its influence on the formation and properties of hydrochar should be further clarified. Hydrochars (HCs), as solid products of hydrothermal treatment, can be used to produce activated carbon and sorbents [54,63,64], supercapacitors [65], humic acids for soil improvement [52,66], wood vinegar [67], or fuel with a higher heating value (HHV) parameter and properties similar to conventional ones [68,69]. Moreover, Zhao et al. [61] underline that hydrochar is a safe solid fuel.

A review of a range of publications related to the pyrolysis of obtained hydrochars proves that biomass after hydrothermal treatment usually emits more volatile pyrolysis products [49,70,71]. Research on the optimization of thermal processing of biomass with respect to the adaptation of technological processes to the principles of circular economy has shown that the addition of organic wastes in the form of expired NSAIDs can reduce the contribution of undesirable compounds in volatile pyrolysis products [72,73]. The experimental data proved that the used additives had different effects on the relative contribution of hydrocarbons in the composition of volatile pyrolysis products [72]. However, in the case of the waste of sunflower inflorescences, which is characterized by a higher content of inorganic components, the addition of 2 wt% of expired paracetamol tablets proved to be more effective in reducing the relative contribution of undesirable hydrocarbons in the composition of products of its pyrolysis [73].

However, one aspect remains unexplained. Despite the widespread statements about carbon neutrality during the energy processing of biomass products, there is a distinct lack of publications that focus on the increase in the amount of environmentally hazardous compounds in volatile products with respect to the emitted amount of CO_2_ (which is possibly absorbed by plants during their growth, in line with the concept of carbon neutrality). Therefore, the aims of this research are

(i) The evaluation of the yield of the products of two biomass materials (sunflower stems and inflorescence) originating from the same plant during autohydrolysis that was carried out at temperatures of 120, 150, and 180 °C;
(ii) The characterization of the evolution of structural and structural–chemical parameters of the obtained products depending on the temperature of autohydrolysis;
(iii) The analysis of the course of the pyrolysis process of the obtained hydrochars combined with a quantitative evaluation of the evolution of the structure of the pyrolyzed hydrochars and a semi-quantitative evaluation of the changes in the composition of volatile pyrolysis products with respect to the emitted CO_2_;
(iv) The investigation of the influence of 2 wt% addition of expired paracetamol on the composition of volatile pyrolysis products obtained from hydrochars and the migration of inorganics during the pyrolysis process.

## 2. Results and Discussions

### 2.1. Influence of Hydrothermal Treatment (Autohydrolysis) on Characteristics of Hydrochars

Table 1 presents the results of the determined yields of the dried hydrochars (HCs), aqueous hydroliquors (AHLs), and gas phase that were obtained during hydrothermal treatment of the sunflower stems (SS) and sunflower inflorescences (SI) samples.

The data in Table 1 imply that during autohydrolysis, more AHLs and less HCs are obtained from the SI sample compared to the SS sample.

Table 2 presents the basic characteristics of the obtained hydrochars, which allows for their comparison with the characteristics of their initial biomass.

It follows from the data in Table 2 that the amount of C atoms increased in the studied samples with an increase in temperature of the hydrothermal treatment; there was a visible tendency for H atoms to increase and for N, S, and O atoms to decrease. The raw SI sample was characterized by a 1.3 times greater amount of ash than that of the raw SS sample. As a result of autohydrolysis at temperatures of 120, 150, and 180 °C, the ash contents of hydrochars of the SS sample decreased by 2.61, 3.28, and 5.28 times appropriately, whereas those of the SI sample decreased by 1.77, 1.82, and 2.23 times, respectively. Therefore, the rate of removal of inorganics from the SI sample during autohydrolysis was lower, but the increase in the HHV parameter was greater due to a higher content of C atoms in the hydrochars. Similar results regarding an increased content of C atoms, a decrease in ash content, and a rise in HHV parameter were discussed also in other publications [74,75]. The application of the XRF technique proved that more and more AAEMs were removed from the SS sample with an increase in autohydrolysis temperature (Table S1). In the case of the SI sample, the removal of some elements was selective, which comes in good agreement with previous opinions [30,44,76]. Klasson et al. [76] pointed out that the removal of AAEMs from biomass by washing with water had a limited effect; it was impossible to remove Ca and Fe atoms completely. The authors of [30,44] generally believed that heavy metals were retained in hydrochars during hydrothermal treatment.

The results presented in Table 1 and Table 2 give grounds to suggest that the studied SS and SI samples are characterized not only by distinct amounts of ash and dynamics of removal of inorganics during autohydrolysis but also by different solubilities in hot liquid water and probably divergent compositions and structures.

### 2.2. TG Test of SS and SI Samples and Their HCs

**Figure 1:**
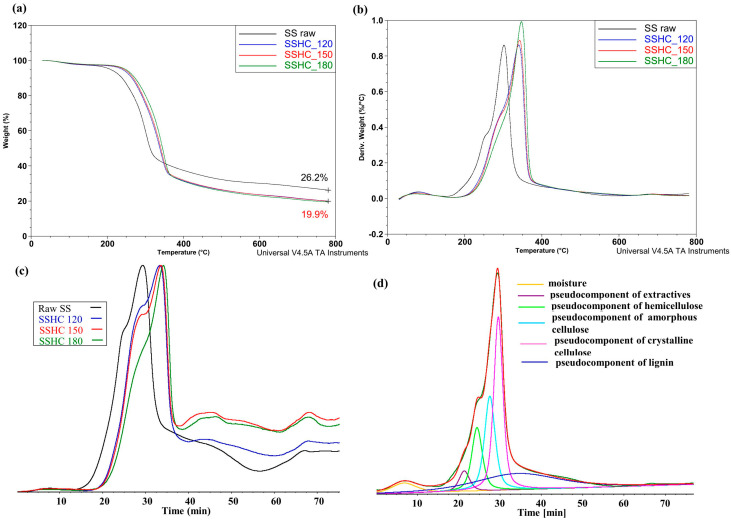
The curves of weight loss (**a**), weight loss rate (**b**), and the Gram–Schmidt (G-S) curves (**c**) registered during pyrolysis of SS sample and its HCs, and the results of the deconvolution of the DTG curve of the raw SS sample (**d**).

Figure 1 presents the curves of weight loss, weight loss rate, and the Gram–Schmidt (G-S) curves of raw SS sample and its HCs at different autohydrolysis temperatures, and the results of the deconvolution of the DTG curve of the raw SS sample.

The comparison of the shapes of the TGA curves in Figure 1a implies that, after autohydrolysis, the TGA curves of the SSHC samples shift towards higher temperatures. The removal of components soluble in hot liquid water from the raw SS sample reduced the yield of solid pyrolyzed material from all HCs at a temperature above 350 °C in the same way. A similar effect of reduced yield of HCs was described in the works by other authors [56,70,77,78]. In the publication by da Silva et al. [71], despite the shift in the TGA curves of wood and bark towards higher temperatures, there was a higher yield of pyrolyzate of HC from wood and a lower yield of pyrolyzate from bark ascertained in comparison to the samples of pyrolyzates not treated with water.

The DTG curves of SSHCs also shifted towards higher temperatures, implying that their decomposition starts at higher temperatures than that of the raw SS sample (Figure 1b). The shape of the DTG curves of SSHCs suggests that a component involved in the formation of DTG curves at lower temperatures was removed from the raw SS sample during autohydrolysis, or the physicochemical properties of this component changed towards higher thermal stability. A similar effect of the shift in DTG curves of HCs towards higher temperatures was observed by other researchers, who suggested that this shift may have been caused by the dissolution of non-structural components present in biomass (e.g., hemicellulose) and by the partial dissolution of polysaccharides and lignin [79,80].

It follows from Figure 1c that the shape of the G-S curves of hydrochars changed in comparison with the corresponding curve of the raw SS sample, and the peaks shifted by approximately 40 °C towards higher temperatures. The changes in shape of these curves point to the changes in thermal stability of the material of hydrochars and imply the changes in their physico-chemical properties. Figure 1d presents the results of the deconvolution of the DTG curve of the raw SS sample. These results show that in this sample, apart from moisture (5.1%) and extractives (4.2%), 13.4% of the pseudocomponent of hemicellulose, 20.0% of the pseudocomponent of amorphous cellulose, 31.0% of the pseudocomponent of crystalline cellulose, and 26.3% of the pseudocomponent of lignin undergo decomposition during pyrolysis. This means that the pseudocomponent of cellulose was predominant (51%) in the composition of the raw SS sample. The positions of the peaks of the marked pseudocomponents indicate that the temperature peak of the higher decomposition rate of the pseudocomponent of hemicellulose amounts to 240 °C, that of the pseudocomponent of amorphous cellulose to 270 °C, that of the pseudocomponent of crystalline cellulose to 290 °C, and that of the pseudocomponent of lignin to 350 °C. The aforementioned temperatures are higher than those for the components of cotton stalk published by Wang et al. [67] and those for individual components of biomass presented in the work by Yang et al. [81] but are very similar to the temperatures of the decomposition rate of pseudocomponents of biomass given in [82]. This means that various biomass types have different decomposition temperatures of basic components under similar pyrolysis conditions.

**Figure 2:**
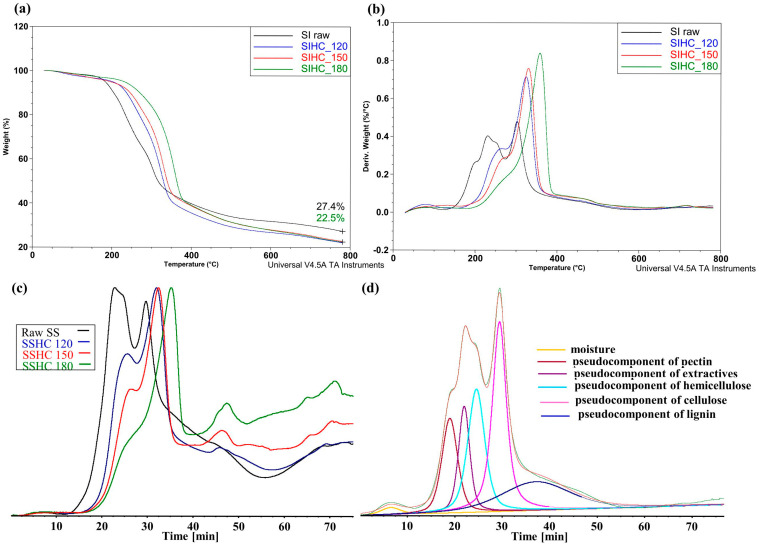
The curves of weight loss (**a**), weight loss rate (**b**), and the Gram–Schmidt (G-S) curves (**c**) registered during pyrolysis of SI sample and its HCs, and the results of the deconvolution of the DTG curve of the raw SI sample (**d**).

Figure 2 presents the thermogravimetric curves, the G-S curves registered during pyrolysis of the SI sample and its HCs, and the results of the deconvolution of the DTG curve from the raw SI sample.

It follows from the shape of the curves of the changes in weight loss depending on temperature presented in Figure 2a that, compared to the raw SI sample, the thermal decomposition of SIHCs starts at higher temperatures. The delayed onset of the decomposition of SIHCs is consistent with the rise in autohydrolysis temperature. The hydrothermal treatment causes the weight loss of pyrolyzed HCs in the temperature range of 200–375 °C in different ways. In the temperature range of 375–750 °C, the TGA curves of the SIHC_150_ and SIHC_180_ samples coincided. At the temperature of 750 °C, the weight loss for all HCs is the same, being greater than that of the pyrolyzate from the raw SI sample by almost 5%.

The shape of the DTG curve of the raw SI sample (Figure 2b) implies that there should be a larger number of components present in it than in the raw SS sample. It follows from Figure 2b that autohydrolysis caused not only the removal of a great amount of material in the form of AHLs (Table 1) but also a substantial transformation of the DTG curves. Such dramatic changes in the composition of the sample after autohydrolysis were confirmed by the G-S curves in Figure 2c. Some peaks in these curves not only shifted towards higher temperatures after autohydrolysis but also showed changes in their heights.

It follows from Figure 2a that at the temperature of 300 °C, the pyrolyzed sample of the raw SI sample lost more than 50% of its weight. The deconvolution of the DTG curve of the raw SI sample (Figure 2d) proved that the formation of this curve up to the temperature of 300 °C was mainly predetermined by the pseudocomponent of pectin present in the raw SI sample in the amount of 14.9%, the pseudocomponent of extractives in the amount of 12.4%, and the pseudocomponent of hemicellulose in the amount of 21.6%. At higher temperatures, the course of the DTG curve was mainly connected with the decomposition of the pseudocomponent of cellulose (29.7%) and the pseudocomponent of lignin (19.7%). The comparison of data on the curves in Figure 1d and Figure 2d proved that the studied SS and SI samples differed substantially by the composition of organic material and, according to the data in Table 1 and Table 2, the differences related to both solubility of their organic components in hot liquid water and solubility of inorganics during the process of autohydrolysis.

### 2.3. Analysis of Evolution of Structural–Chemical Parameters of SS and SI Samples Caused by the Increase in Autohydrolysis Temperature

**Figure 3:**
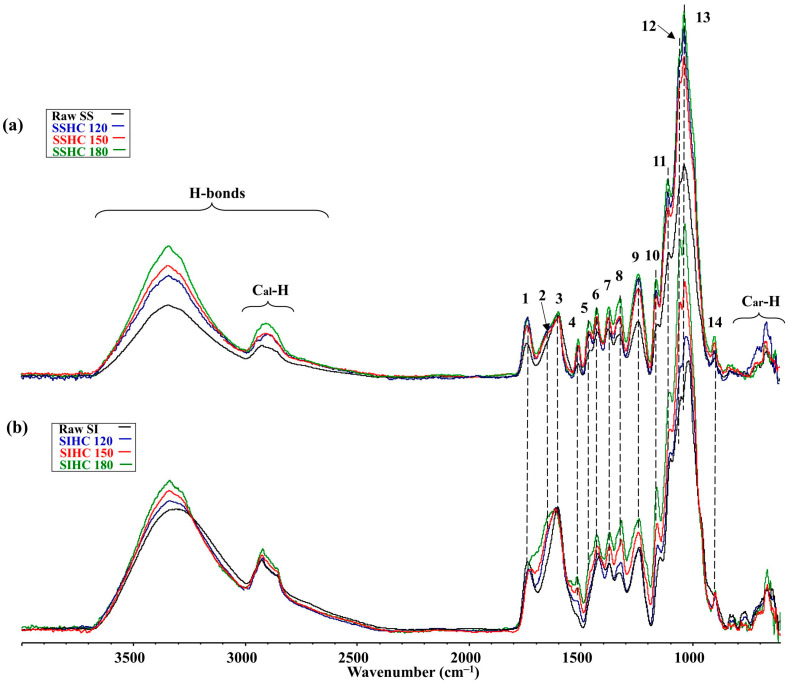
The ATR spectra of the raw SS (**a**) and SI (**b**) samples and their HCs. Assignment of bands: 1—vibration of esters, ketones, and aldehydes; 2—absorbed O–H and conjugated C–O in fragments of lignin or cellulose; 3—aromatic skeletal vibrations; 4—aromatic skeletal vibrations of guaiacyl rings; 5—CH– deformation, asymmetric in plan for lignin; 6—aromatic skeletal vibration combined with CH in-plane deformation for lignin and cellulose; 7—CH deformation in cellulose; 8—C–O vibration in syringyl and guaiacyl rings, C–H cellulose; 9—guaiacyl ring breathing, C–O stretch in lignin; C–O linkage in guaiacyl aromatic methoxyl groups; 10—C–H vibration in lignin and C–O stretching in cellulose; 11—C–O–C glycosidic ether and C–C ring breathing; 12—stretching and bending vibrations of C–O, C–C, C–OH, and glycosidic C–O–C; 13—C–O–C pyranose ring skeletal vibration; 14—C–H out-of-plane glucose ring in cellulose and hemicellulose, and in guaiacyl rings in lignin [82,83,84,85,86,87].

To evaluate the changes in structural–chemical parameters of the SS sample caused by autohydrolysis, it was essential to implement infrared spectroscopy. Figure 3 presents the ATR spectra of the raw SS and SI samples and their HCs that were obtained at different temperatures of autohydrolysis.

The analysis of the shape of the normalized ATR spectra in Figure 3a implies that the removal of soluble organic and inorganic components of the raw SS sample during autohydrolysis caused a visible transformation of bands present in the spectra and, thus, the changes in structural–chemical parameters of HCs that were obtained at different temperatures. Compared to the raw SS sample, the heights of the bands of H-bonds, of bands of the C_al_-H type, of bands of compounds containing C=O groups, and of bands in the range of 1530–1100 cm^−1^ increased for all HCs. In the range of 1530–1100 cm^−1^, the heights of bands tend to increase with a growth in the temperature of hydrothermal treatment from 120 to 180 °C. The contribution of groups of atoms, the presence of which corresponds to bands 1 and 4–14, increases. The spectra presented in Figure 3a imply that, after autohydrolysis of the raw SS sample, the contribution of groups of atoms and bonds characteristic of cellulose and lignin increased with the temperature growth. According to Gandolfi et al. [88], the band near 1740 cm^−1^ was supposed to correspond to the presence of a carbonyl group connected with cellulose. This cannot be fully agreed with because the experimental data suggest that the removal of hemicellulose from the raw SS sample during autohydrolysis should have lowered the height of band 1, whereas this height increased in the spectra of all HCs.

A contrastive analysis of structural–chemical transformations taking place in the raw SI sample and its HCs during autohydrolysis is presented in Figure 3b. Similarly to the SS sample and its HCs (Figure 3a), after autohydrolysis, the SI sample shows a tendency towards the increased contribution of bands from H-bonds of all HCs in the formation of the ATR spectra, but the extent of these changes is smaller. In the ATR spectra, the heights of 4–13 bands within the fingerprint range of 1550–1000 cm^−1^ (Figure 3b) increased, and the bands became more visible after the dissolution of non-structural components. In contrast to the SSHC samples, no increase in heights of the bands corresponding to the presence of compounds with carbonyl groups was observed in hydrochars of the SIHC samples.

**Figure 4:**
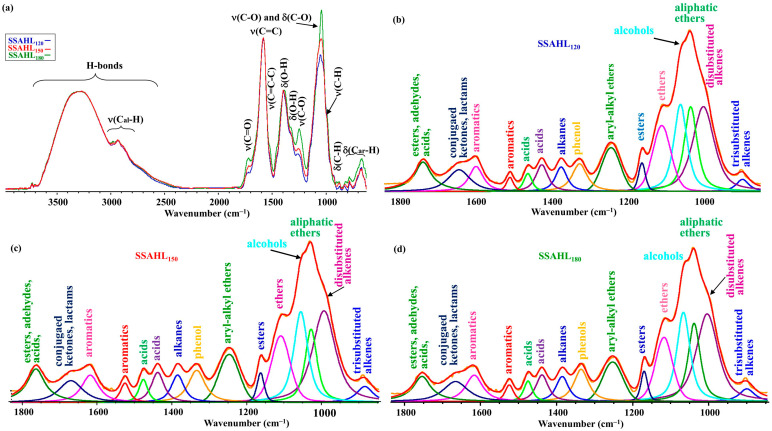
The ATR spectra of SSAHLs obtained during autohydrolysis of the raw SS sample (**a**), the deconvolution of a fragment of the ATR spectrum of SSAHL_120_ (**b**), the deconvolution of a fragment of the ATR spectrum of SSAHL_150_ (**c**), and the deconvolution of fragment of the ATR spectrum of SSAHL_180_ (**d**) (assignment of bands according to [89,90,91,92]).

Figure 4 presents the ATR spectra of SSAHLs obtained during autohydrolysis.

The ATR spectra of the SSAHL samples within the fingerprint range presented significantly fewer bands compared to the spectra of hydrochars of the SS sample (Figure 4a). All normalized spectra of AHLs had the same heights of bands of H-bonds. The band near 1700 cm^−1^ corresponds to the presence of compounds with carbonyl groups. The results of the research presented in [81,87,93,94,95] give grounds to suggest that this band corresponds to the presence of esters, aldehydes, acids, and ketones [89,90,91,92]. The height of this band increased with a growth in temperature. The deconvolution in the fingerprint range of the ATR spectra presented in Figure 4b–d implies the presence of di- and trisubstituted alkenes, alcohols, phenols, ethers, alkanes, and esters in SSAHLs. The calculations of the sub-peak surfaces of these bands, which characterize their relative contribution to the creation of the fingerprint range spectrum, indicate that the amount of these compounds in SSAHLs does not practically change with the increase in autohydrolysis temperature (Table S2). The temperature of 180 °C did not influence the contribution of ν(C_al_-H) bands in the formation of the ATR spectrum but increased the contribution of bands of the δ(C_ar_-H) type in the range of 900–600 cm^−1^.

**Figure 5:**
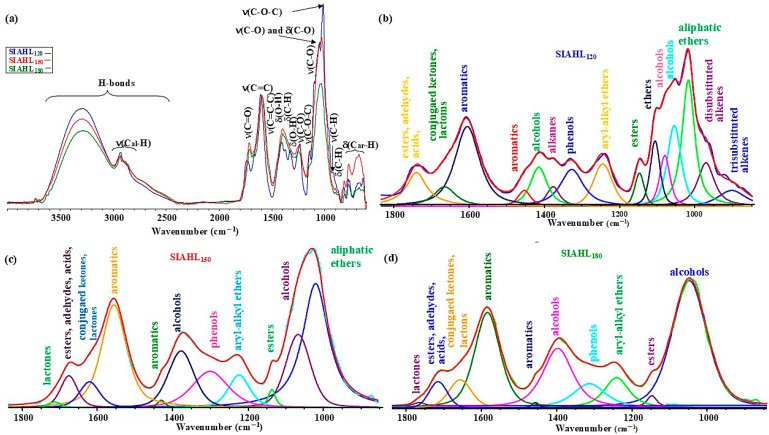
The ATR spectra of SIAHLs obtained during autohydrolysis of the raw SI sample (**a**), the deconvolution of a fragment of the ATR spectrum of SIAHL_120_ (**b**), the deconvolution of a fragment of the ATR spectrum of SIAHL_150_ (**c**), and the deconvolution of a fragment of the ATR spectrum of SIAHL_180_ (**d**) (assignment of bands according to [89,90,91,92]).

The analysis of the ATR spectra of SIAHLs presented in Figure 5 supplements the re-distribution of groups of atoms and functional groups during autohydrolysis of the SI sample.

The contrastive analysis of the spectra in Figure 4 and Figure 5 suggests that the material of SIAHLs, which was formed during autohydrolysis of the raw SI sample, had a completely different course of structural–chemical transformation and showed the opposite nature of changes in the heights of the bands depending on the autohydrolysis temperature to that of the material obtained from the raw SS sample after autohydrolysis. Namely, there was a significant decrease in the heights of the bands of H-bonds and bands of ν(C–O–C), ν(C–O) and δ(C–O) (the range of 1075–1020 cm^−1^) with the rise in autohydrolysis temperature. This implies that an increase in autohydrolysis temperature above 120 °C lowered the contribution of polar compounds in hot liquid water, which can result from the activation of reactions between polar compounds with the rise in temperature of hot liquid water. In the range of 1700–1100 cm^−1^, the heights of the ν(C–O), δ(C-H), δ(O–H), and ν(C–O–C) bands from the SIAHL_150_ sample become greater, just like those in the range of 900–600 cm^−1^ for the bands of bending out of plane δ(C_ar_-H) from the SIAHL_150_ sample. The calculations of sub-peaks obtained during deconvolution (Figure 5b–d) imply that the increase in autohydrolysis temperature causes an increase in relative contribution of the bands of alcohols, phenols, and aromatics and a decrease in relative contribution of ethers, alkanes, and compounds with carbonyl groups (Table S2).

The evolution of structural–chemical parameters of the material of HCs and the material removed from biomass in the form of AHLs implies that a reorganization of the structure of the raw SS sample occurs during autohydrolysis. Such reorganization can be traced with the use of corresponding diffractograms of HCS.

**Figure 6:**
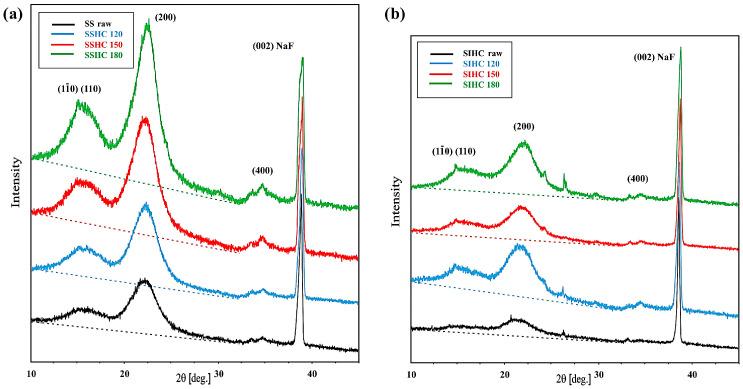
The diffractograms of raw SS (**a**) and SI (**b**) samples and their HCs.

Figure 6 presents the diffractograms of the raw SS (Figure 6a) and SI (Figure 6b) samples and their HCs.

The diffractograms clearly present the reflexes of coherent scattering that are characteristic of crystalline cellulose: the superposition of the (1$\bar{1}$0), (110), and (200) reflexes and of the (400) reflex. In the diffractograms, the background lines, which correspond to the intensity of non-coherent scattering from the non-ordered fragments of the structure (amorphous), are marked. It follows from the profiles of the reflexes of SSHCs that the integral intensity of the (200) reflex and overlapping (1$\bar{1}$0) and (110) reflexes of cellulose increases with the growth in autohydrolysis temperature. The ratio of integral intensity of the discussed reflexes to the integral intensity of the (002) reflex from NaF show an increase in height of the reflex and a decrease in its half-width, pointing to a higher degree of ordering of cellulose chains in HCs. The values of this increase in ratios of integral intensity for the (1$\bar{1}$0) and (110) reflexes with the rise in autohydrolysis temperature change in a sequence of 1.05, 1.78,1.86, and 2.27, while those for (200) reflex, 3.57, 5.36, 5.60, and 5.63 (Table S2). The effect of structure ordering of HCs was considered by the authors of works [83,96] as an increase in crystallinity index in hydrochars. The increase in ordering degree of cellulose chains facilitated their thermal stability and resulted in a shift in peaks on the DTG and G-S curves of hydrochars towards higher temperatures (Figure 1).

The differences in dependence of the contribution of H-bonds in the formation of the ATR spectra of SIAHLs on the temperature (Figure 5a) could have resulted in a distinct course of structural reorganization of SIHCs during autohydrolysis. Figure 6b presents the diffractograms of the raw SI sample and its HCs.

It follows from Figure 6b that the transformation in the shape of the diffractograms of the SIHC samples with an increase in autohydrolysis temperature substantially differs from that observed for SSHCs in Figure 6a. The profile of the background line from SIHC_120_ is inclined at a greater angle with respect to the background line of the raw SI sample and its other HCs. The significant raising of the background line of the SIHC_120_ sample from lower angles of scattering implies a greater amount of non-ordered material in it, which causes the occurrence of incoherent X-ray scattering. This may have been connected with the presence of some amount of polar compounds in the SIHC_120_ sample remaining after the removal of SIAHL_120_, which was characterized by a great amount of polar compounds able to form hydrogen bonds. This increase may have happened because the HCs were not additionally rinsed with water after autohydrolysis but only separated on a Buchner funnel. Small amounts of this material could have been an obstacle in the process of ordering of cellulose chains. A higher autohydrolysis temperature could have eliminated this obstacle and initiated the processes of ordering of cellulose. Nevertheless, the calculations of the ratios of the integral intensity of reflexes that resulted from coherent scattering and the (002) reflex from the internal standard NaF point to an increase in the ordering of cellulose chains. The integral intensity of reflex that resulted from the superposition of the (1$\bar{1}$0) and (110) lines in the SIHC_120_ sample increased by almost twofold. The ratios of integral intensities of the (1$\bar{1}$0) and (110) reflexes to the integral intensity of the reflex from NaF vary in a sequence of 0.48, 0.89, 0.94, and 1.25 (Table S3). The ratio of integral intensity of the (200) reflex to the integral intensity of the reflex from NaF of all HCs also increases with the rise in autohydrolysis temperature in a sequence of 1.72, 2.98, 3.13, and 3.97, as presented in Table S3 and Figure 6b.

The research results presented above imply that the evolution of the structure of the products of autohydrolysis of the SS and SI samples occurs in different ways and the level of observed changes is not proportional to the changes in amount of inorganic and organic components removed from the samples during autohydrolysis.

### 2.4. Analysis of the Influence of Changes in Autohydrolysis Temperature on the Composition of Volatile Pyrolysis Products of Obtained HCs

**Figure 7:**
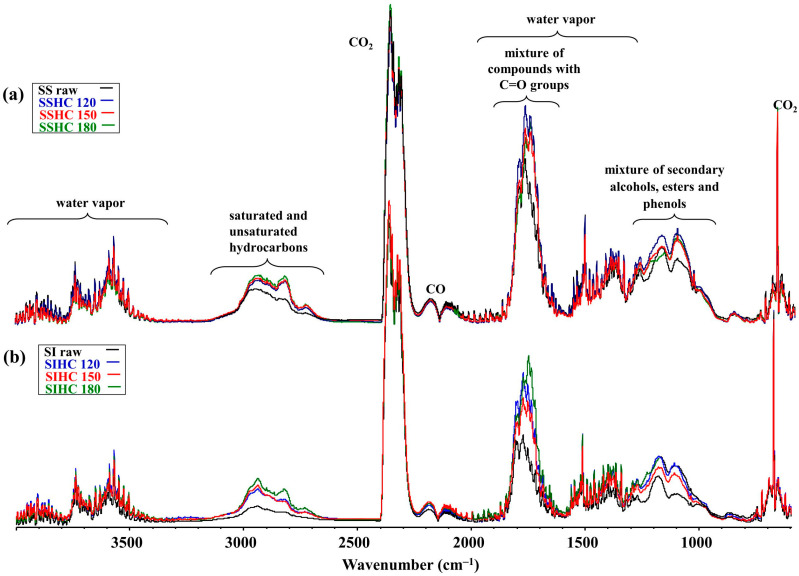
The FT-IR spectra of the volatile pyrolysis products of raw SS (**a**) and SI (**b**) samples and their HCs.

Figure 7 presents the FT-IR spectra of the volatile pyrolysis products of raw SS and SI samples and their HCs at the temperature corresponding to the maximum of weight loss rate.

It follows from the analysis of the shapes of the spectra in Figure 7a that all samples of SSHCs have a similar shape of normalized bands. This implies that the composition of volatile pyrolysis products emitted from SSHCs is similar. The ratios of bands of saturated hydrocarbons during pyrolysis of SSHCs and the raw SS sample calculated by OMNIC9.2.86 software (Tables S4 and S5) prove that the SSHC_120_, SSHC_150_, SSHC_180_ samples during pyrolysis at the temperature of maximum weight loss rate emit 1.70, 1.74, and 1.86 times more saturated and unsaturated hydrocarbons correspondingly than the raw SS sample. The emission of compounds with carbonyl groups in a series of increasing temperatures of autohydrolysis changed in a sequence of 1.39, 1.24, and 1.28. With an increase in autohydrolysis temperature, the contribution of phenols in the composition of volatile pyrolysis products decreases in a sequence of 1.56, 1.47, and 1.42. For alcohols, this sequence changed with a rise in autohydrolysis temperature as follows: 1.25, 1.14, and 1.13. The presented numerical values indicate that during pyrolysis of hydrochars, the concentration of compounds with carbonyl groups, alcohols, and phenols tends to decrease in the composition of volatile pyrolysis products with the increase in autohydrolysis temperature.

The changes in ash content in SSHCs (Table 2) as well as the rate of removal of AAEMs (Table S1) with the increase in autohydrolysis temperature are not proportional to the weight loss of hydrochars (Figure 1a) and to the calculations of changes in surface ratios of bands (Tables S4 and S5) in the spectra in Figure 7a. The lack of proportionality gives grounds to suggest that the removal of inorganics does not noticeably influence the weight loss and the yield of volatile products of the pyrolysis of hydrochars. This suggests that other factors may have influenced the yield of volatile pyrolysis products, primarily the absence of extractives and hemicelluloses in the hydrochar composition.

The investigations carried out by a range of researchers [97,98,99] proved that there are some interactions taking place between cellulose, hemicellulose, and lignin that affect the yield and composition of volatile products of pyrolysis. The increasing removal of hemicellulose and extractives during the rise in autohydrolysis temperature consequently shifts the peaks in the G-S and DTG curves towards higher temperatures and eliminates the influence of volatile products of pyrolysis of hemicellulose on cellulose and lignin. According to the data obtained by Usino et al. [100], pyran and furan compounds as well as reactive minor and major light oxygenated compounds are formed during the pyrolysis of xylan as one of the components of hemicellulose. Similar compounds that are present in the volatile products of dehydration, depolymerization, and rearrangement of hemicellulose in raw biomass during pyrolysis can modify the other components of biomass, cellulose and lignin. The vanishing presence of hemicellulose and pectin in hydrochars eliminates their influence on cellulose and lignin and changes the composition and yield of volatile products of pyrolysis of these HCs.

**Figure 8:**
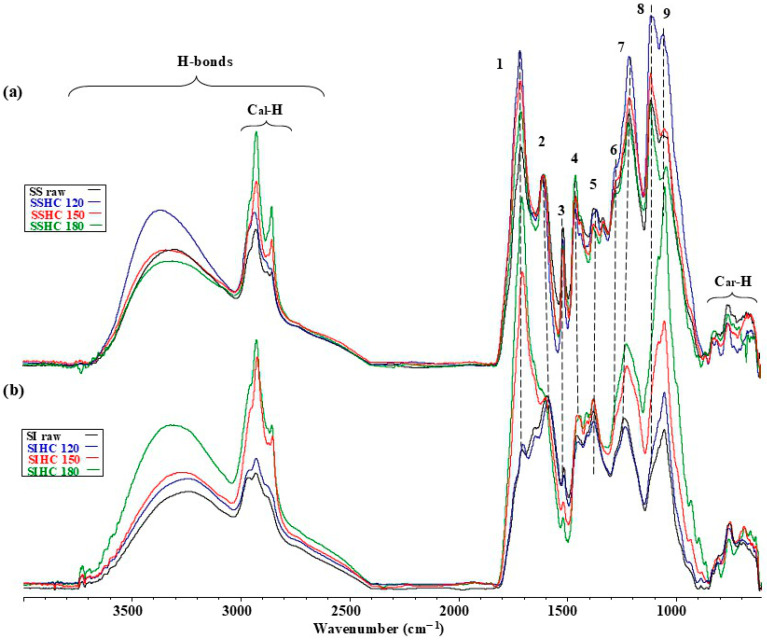
The spectra of condensed material from volatile products of pyrolysis of raw SS (**a**) and SI (**b**) samples and their HCs. Assignment of bands: 1—vibration of esters, ketones, and aldehydes; 2—absorbed O–H and conjugated C–O in fragments of lignin or cellulose; 3—aromatic skeletal vibration; 4—aromatic skeletal vibrations guaiacyl rings; 5—CH– deformation, asymmetric in plan for lignin and hemicellulose; 6—aromatic skeletal vibration combined with CH in-plane deformation for lignin and cellulose; 7—CH deformation in cellulose and hemicellulose; 8—guaiacyl ring breathing, C–O stretch in lignin; C–O linkage in guaiacyl aromatic methoxyl groups, C–O stretch in syringyl rings; 9—stretching and bending vibrations of C–O, C–C, C–OH, and the glycosidic C–O–C [82,83,84,85,86,87].

The specificity of transformations of structural–chemical parameters and the ordered structure of HCs could have affected the thermal behavior of the pyrolyzed SIHC samples. Figure 8b presents the normalized FT-IR spectra of volatile products of pyrolysis of the raw SI sample and its HCs at different autohydrolysis temperatures. In the normalized FT-IR spectra of volatile products (Figure 7b), the bands of saturated and unsaturated hydrocarbons are marked in the wavenumber range of 3200–2600 cm^−1^. The normalized surface ratios of these bands of SIHC_120_, SIHC_150_, and SIHC_180_ to the analogous band of raw SI sample increased by 1.94, 2.13, and 2.74 times, correspondingly. The surface ratios of bands of a mixture of compounds with carbonyl groups in the spectra of hydrochars to the surface of corresponding bands in the spectrum of the raw SI sample changed in a sequence of 1.39, 1,69, and 1,85. For the bands from alcohols, this sequence was 1.25, 1.58, and 1.65, and for phenols, 1.49, 1.83, and 1.85 (Tables S4 and S5). The presented numerical values imply that the contribution of saturated and unsaturated hydrocarbons, the mixture of compounds with carbonyl groups, alcohols, and phenols increased with autohydrolysis temperature in the composition of volatile products of the pyrolysis of hydrochars of the SI sample.

### 2.5. Investigation of Pyrolysis Products of SS and SI Samples and Their HCs Obtained in a Tube Furnace

The differences in the course of evolution of the composition of volatile products of pyrolysis of HCs obtained at different autohydrolysis temperatures are confirmed by the investigations of compounds condensed in methanol. This material was obtained during pyrolysis of the raw SS and SI samples and their HCs in a tube furnace (Figure 8).

The amount of condensates increased with the rise in autohydrolysis temperature from 2.3% for the raw SS sample to 2.8% for the SSHC_120_ sample, 3.0% for the SSHC_150_ sample, and 3.1% for the SSHC_180_ sample, correspondingly. A significant surge in yield of condensed material was obtained during pyrolysis of the SSHC_120_ sample. The spectra of condensates that were obtained during pyrolysis of the raw SS sample and its hydrochars are presented in Figure 8a.

The spectra presented in Figure 8a differ by shape but clearly indicate the dependence of the heights of bands of HCs from autohydrolysis temperature. The heights of the bands from H-bonds and the 1, 4, and 6–9 bands in the spectra of HCs decrease with an increase in autohydrolysis temperature. The increase in autohydrolysis temperature caused a greater degradation of the glycosidic C–O–C band. The exception is the C_al_-H bands in the wavenumber range of 2950–2850 cm^−1^, for which an increase in height is observed with the rise in pyrolysis temperature. This implies that, during pyrolysis of the SSHC_120_ sample, the amount of polar compounds able to form a greater amount of hydrogen bonds increased in the composition of condensed products. The contribution of these compounds in the formation of ATR spectra of the condensates decreased during pyrolysis of the SSHC_150_ and SSHC_180_ samples.

The occurrence of changes in composition of volatile products from the SIHC samples (Figure 8b) was confirmed by the results of determination of the amount of material condensed in methanol, the amount of which increased from 2.3% (raw SI sample) to 4.4% (SIHC_120_), 4.6% (SIHC_150_), and 7.5% (SIHC_180_). These changes in yield of the condensed material imply that, during autohydrolysis of the SI sample at temperatures of 120–180 °C, some reactions between the compounds soluble and insoluble in hot liquid water may have occurred and changed their composition and concentration. This is confirmed by the results of the deconvolution and calculation of the surfaces of sub-peaks in the fingerprint range of ATR spectra of AHLs (Figure 5b–d).

It follows from the ATR spectra of the condensed material (Figure 8b) that the rise in autohydrolysis temperature of the raw SI sample up to the level of 180 °C facilitated an increase in the contribution of polar compounds in the composition of this material and a greater amount of derivative fragments during the decomposition of lignin and cellulose. The heights of H-bond, C_al_-H, and other bands (1, 5–9) in the ATR spectra increased with autohydrolysis temperature. This indicates that the rise in autohydrolysis temperature made more and more biomass material decompose and dissolve in hot liquid water. The increase in the heights of bands 1, 7, and 9 implied that lignin decomposed more actively, which was confirmed by the results obtained by other authors [80,83,97].

The changes in surface ratio of bands in the FT-IR spectra of volatiles (Figure 7) suggest the occurrence of changes in the concentration of compounds with carbonyl groups, alcohols, and phenols with the rise in temperature that can result from the reactions taking place in the aqueous environment during autohydrolysis, which led to the structural–chemical transformations of the parameters of HCs (Figure 3) and the changes in ordering of their structure (Figure 6). The removal of volatiles in the amount of approximately 70% of the material from the raw SS sample and its HCs along with a greater degradation of macromolecules of cellulose (Figure 7) could have caused the changes in the structure of the pyrolyzates.

The inorganics were partly removed from the raw SI sample during autohydrolysis, what was indicated by the ash content in the obtained hydrochars. The content of ash decreased in SIHC_120_ by 1.78 times, in SIHC_150_ by 1.82 times, and in SIHC_180_ by 2.22 times (Table 2). These changes in ash content, which pointed to the concentration of inorganics in SIHCs, were not proportional to the changes in yield and composition of the volatile products of pyrolysis in Figure 7b and to the changes in heights of bands in the ATR spectra of condensates (Figure 8b) obtained during pyrolysis of the SIHC samples. Therefore, it can be presumed that, during autohydrolysis, the removal of non-structural organic components soluble in hot liquid water, which are deprived of participation in the process of pyrolysis of hydrochars, seems to also be the probable reason for the changes in yield and composition of volatile pyrolysis products.

**Figure 9:**
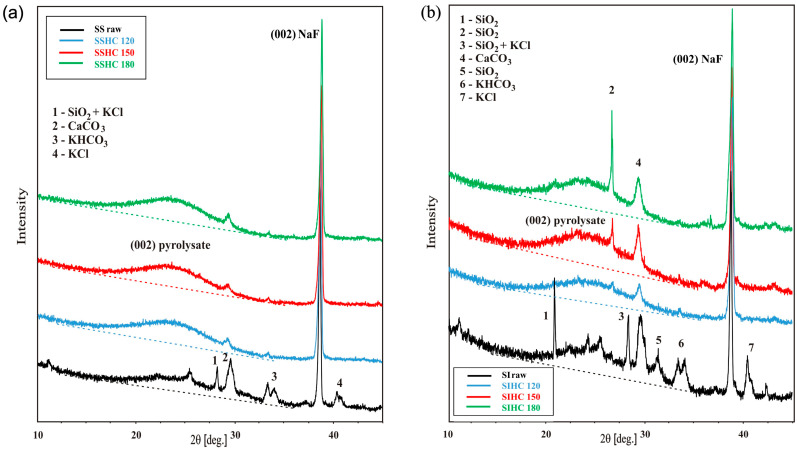
Diffractograms of the pyrolyzed samples of raw SS (**a**) and SI (**b**) samples and their HCs.

Figure 9 presents the diffractograms of the raw SS and SI samples and their HCs pyrolyzed at the temperature of 450 °C.

Compared to the diffractogram of the pyrolyzate of the SS sample, the diffractograms of the pyrolyzed SSHCs did not show too many reflexes originating from inorganics (Figure 9a). The shape of the diffractograms confirmed the removal of inorganics during autohydrolysis. The ordering degree of pyrolyzates, which was evaluated by the ratio of integral intensity of the (002) reflex of every pyrolyzate to the integral intensity of the (002) reflex from NaF, increased with the rise in autohydrolysis temperature in a sequence of 0.83, 1.89, 2.26, and 2.39 (Table S3). The ordering, which is characteristic of crystalline cellulose, disappeared in the pyrolyzed samples at the temperature of 450 °C.

It follows from the diffractograms of the pyrolyzed samples in Figure 9b that the pyrolyzate of the raw SI sample had many reflexes originating from inorganics. The pyrolyzed SIHC samples had significantly fewer reflexes because of their removal during autohydrolysis. The SIHC_180_ sample, from which too many soluble organic components were removed during autohydrolysis, had somehow greater intensity of reflexes from inorganics. Compared to the pyrolyzates of the SSHC samples, the pyrolyzates of the SIHC sample show a greater ordering that was evaluated on the basis of calculations of the ratios of integral intensity of the (002) reflex of pyrolyzates to the integral intensity of the (002) reflex from NaF. The values of these ratios for the pyrolyzed SI sample and SIHCs are 1.32, 2.25, 2.41, and 2.73, correspondingly (Table S3).

### 2.6. Microanalysis of the Composition of Inorganics in Pyrolyzed SS and SI Samples and Their HCs Obtained at the Temperature of 750 °C

The data presented above show that the evolution of structure and structural–chemical parameters of the products with the rise in autohydrolysis temperature of the studied samples takes place differently. To explain the reasons for the observed differences, the SEM/EDS techniques were implemented.

**Figure 10:**
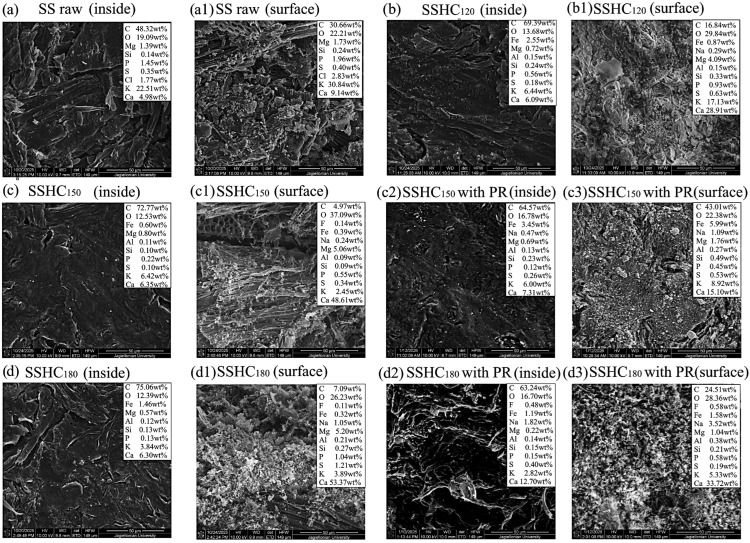
SEM visualization and EDS microanalysis results of the pyrolyzed raw SS sample and its HCs (explanation in the text).

**Figure 11:**
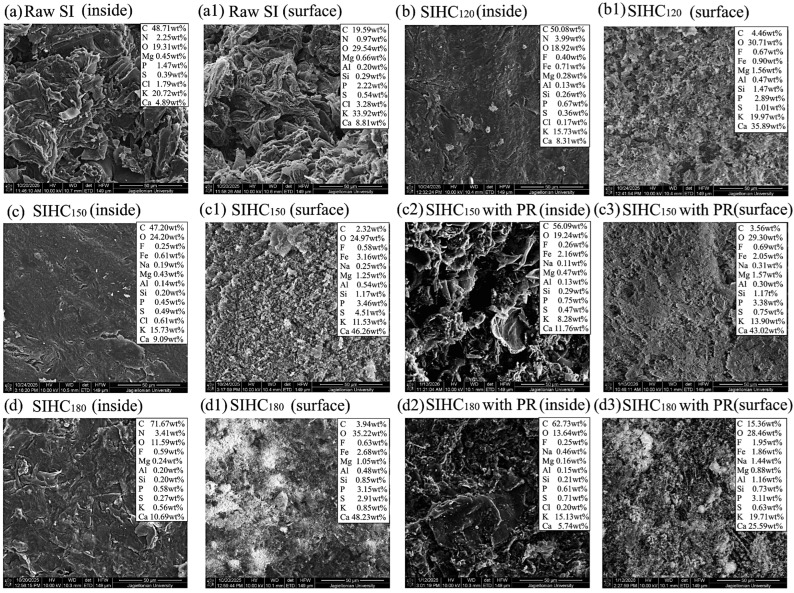
SEM visualization and EDS microanalysis results of the pyrolyzed raw SI sample and its HCs (explanation in the text).

The dynamics of changes in the concentration of AAEMs in the samples of HCs pyrolyzed at the temperature of 750 °C are reflected by the SEM visualization and the results of the EDS microanalysis presented in Figure 10 and Figure 11.

Compared to the pyrolyzed raw SS sample, the results of changes in concentration of elements in pyrolyzed SSHCs, which are presented in Figure 10, are caused not only by the removal of inorganics and the elimination of non-structural components of biomass during autohydrolysis but also by the migration of inorganics in the tablets, their participation in secondary reactions taking place in the gas phase and in the interactions char-volatiles, the removal with volatile pyrolysis products, etc. A complex character of transformations can only allow us to suggest that there is a tendency in the nature of changes in the concentration of inorganics inside of the tablets and on its surface depending on autohydrolysis temperature. It follows from a comparison of the data in Figure 10a–d that the rise in autohydrolysis temperature makes the tendency for a decreasing concentration of Mg (in a sequence of 1.39, 0.72, 0.80, and 0.57 wt%) and K (in a sequence of 2.51, 6.44, 6.42, and 3.84 wt%) and an increasing concentration of Ca (4.98, 6.09, 6.42, and 6.30 wt%) inside of the material of pyrolyzed tablets more visible. The data in Figure 10(a_1_–d_1_) imply that AAEM elements migrating to the surface of pyrolyzed samples show a tendency of decreasing concentrations of K (30.84, 17.13, 2.45, and 3.89 wt%) and increasing concentrations of Mg (in a sequence of 1.73, 4.09. 5.06, and 5.20 wt%) and Ca (in a sequence of 9.14, 28.91, 48.61, and 53.37 wt%) in sediments with the rise in autohydrolysis temperature. According to many authors, the presence of sediments on the surface of pyrolyzates, which contain AAEMs, can affect the secondary reactions taking place between volatile pyrolysis products and the interactions between volatiles and chars at higher temperatures [101,102,103]. Moreover, the CO band visible in the FT-IR spectra of volatile products of pyrolysis (Figure 7) implies that there is a possibility of the Bouduoard reaction between the C on the surface of pyrolyzed hydrochars and the CO_2_ present in volatile products [103]. This reaction can decrease the concentration of C, the tendency of changes for which is visible in Figure 11. Due to the complex course of a series of reactions taking place on the surface of pyrolyzed SSHCs and the results of the EDS microanalysis, it is impossible to determine the dependencies between the concentration of AAEMs in pyrolyzed hydrochars and the nature of the changes taking place in the composition of volatile pyrolysis products in a precise manner.

Figure 11 contains the data confirming the tendency of removal of AAEM elements during autohydrolysis, which occurs against the background of removal of a greater amount of non-structural components from SI samples compared to SS samples (Table 1).

The results of the microanalysis prove that the concentration of K decreases (in a sequence of 20.72, 15.73, 15.73, and 0.56 wt%) inside of the tablets of pyrolyzed SIHCs with the rise in autohydrolysis temperature, whereas the concentration of Ca increases (4.89, 8.31, 9.09, and 10.69 wt%, respectively) (Figure 11a–d). A tendency similar to that presented by the data in Figure 11(a_1_–d_1_) is observed on the surface of pyrolyzed tablets: the concentration of K in sediments decreases in a sequence of 33.92, 19.97, 11.53, and 0.85 wt%, while the concentration of Ca increases in a sequence of 8.81, 35.89, 46.26, and 48.23 wt% with the rise in autohydrolysis temperature.

The data presented above show that, with increasing autohydrolysis temperature inside of pyrolyzed tablets of HCs, the amount of K and Mg decreases and that of Ca increases. However, the concentration of K decreases (by 8 times for SSHC_180_ and almost by 40 times for SIHC_180_) in the sediment on the sample surface, while those of Mg and Ca increase. This increase is 3-fold for Mg and 5.8-fold for Ca on the surface of the pyrolyzed SSHC_180_ sample, and 5.5-fold for Ca on the surface of the pyrolyzed SIHC_180_ sample. The negative changes in concentration of AAEMs in the sediment of pyrolyzed HCs implies a lack of migration of K with volatile pyrolysis products to the surface of samples. The presented tendency suggests a possible positive influence of K on the course of secondary reactions in volatile pyrolysis products, which could have led to a reduction in undesirable compounds in their composition. However, the increasing migration of Mg and Ca tends to cause undesirable changes in the composition of volatiles.

### 2.7. Influence of PR Additive on the Pyrolysis Processes of SSHC and SIHC Samples and on the Composition of Their Volatile Pyrolysis Products

**Figure 12:**
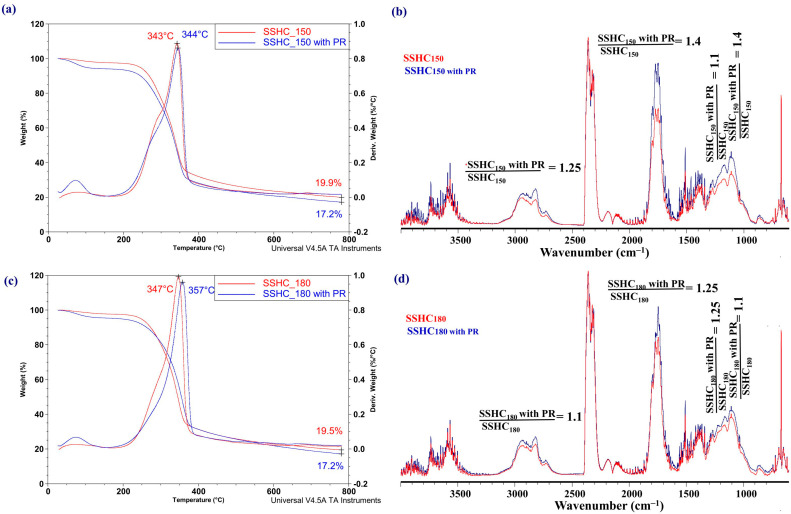
The TGA and DTG curves of the SSHC_150_ (**a**) and SSHC_180_ (**c**) samples, the FT-IR spectra of volatile products of their pyrolysis, and the blends of these samples with PR (**b**,**d**).

Figure 12 presents a comparison of the TGA and DTG curves, normalized FT-IR spectra of the pyrolysis products of the SSHC_150_ and SSHC_180_ samples, and their blends with PR.

The comparison of the shapes of the curves in Figure 12a,c suggests that the addition of 2 wt% of PR increases the weight loss rate of pyrolyzed SSHC samples, increases their weight loss rates, and shifts the temperatures of the maximum weight loss rate towards higher values. During pyrolysis of the blends of SSHC_150_ and SSHC_180_ samples with PR, the contribution of saturated and unsaturated hydrocarbons increases by 1.25 and 1.1 times, respectively; compounds with carbonyl groups by 1.4 and 1.25 times; phenols by 1.1 and 1.25 times; and alcohols by 1.4 and 1.1 times in the composition of their volatile pyrolysis products (Figure 12b,d). There is a visible tendency to reduce the negative effect of PR addition on the relative contribution of undesirable compounds in the composition of volatile pyrolysis products of HCs obtained at a higher autohydrolysis temperature (Tables S6 and S7).

**Figure 13:**
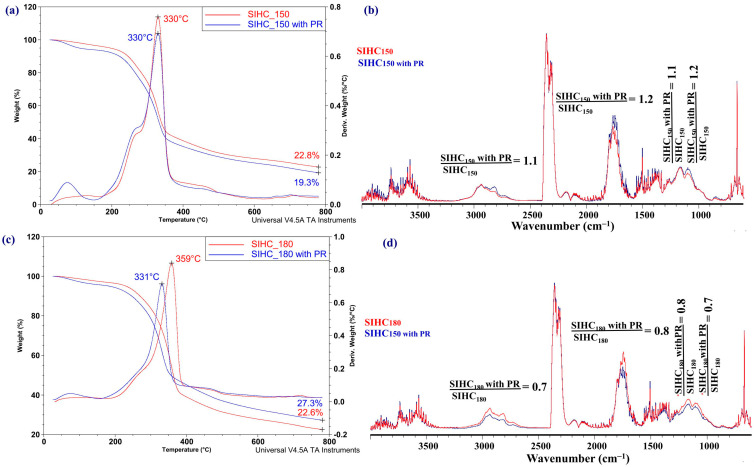
The TGA and DTG curves of the SIHC_150_ (**a**) and SSHC_180_ (**c**) samples, the FT-IR spectra of volatile products of their pyrolysis, and the blends of theses samples with PR (**b**,**d**).

A distinct situation is observed in case of the blends of SIHCs with the PR additive (Figure 13).

The shape of the curves in Figure 13a,c proves that the addition of PR increases the weight loss rate during pyrolysis of the SIHC_150_ sample but decreases it for the SIHC_180_ sample, and does not change the temperature of the maximum weight loss rate for the SIHC_150_ sample but decreases it for the SIHC_180_ sample. For both samples, the maximum value of the derivative weight loss rate decreases.

It follows from the comparison of the shape of the FT-IR spectra registered at the temperature of the maximum weight loss rate that the addition of 2 wt% of PR causes a slight increase in the contribution of saturated and unsaturated hydrocarbons, compounds with carbonyl groups, alcohols, and phenols in the composition of volatile pyrolysis products of the SIHC_150_ (Figure 13b) sample and a decrease in the contribution of these compounds in the volatile pyrolysis products of the SIHC_180_ sample (Figure 13d).

The data presented in Figure 12 and Figure 13 imply that the increase in relative contribution of undesirable compounds in the composition of volatile pyrolysis products of HCs obtained at higher autohydrolysis temperatures can be reduced by the addition of 2 wt% of expired paracetamol. It cannot be excluded that other additives of waste materials, which are able to exert influence on the reactions of hydrogen disproportionation in carbon-based materials, can similarly alter the composition of volatile pyrolysis products. However, additional research is required to confirm this supposition.

To evaluate the influence of paracetamol on the migration of inorganic substances during the pyrolysis of HCs, their concentration was compared in the samples pyrolyzed with and without the addition of this waste. Compared to the pyrolyzed SSHC_150_ samples, the addition of 2 wt% of paracetamol caused changes in the contribution of inorganics inside of the pyrolyzed tablets.

The concentration of Mg decreased from 0.80 to 0.69 wt%, the concentration of K reduced from 6.42 to 6.00 wt%, but the concentration of Ca increased from 6.35% to 7.31 wt% (Figure 10c,c_2_). However, the composition of sediment on the pyrolyzed SSHC_150_ tablet changed significantly. The contribution of K increased almost by 3.5 times, while the contribution of Ca and Mg decreased by almost 3 times (Figure 10(c_1_,c_3_)). Inside of the pyrolyzed SSHC_180_ samples with PR, the contribution of Ca almost doubled, the contribution of K decreased by 1.4 times, and the contribution of Mg decreased by 2.6 times (Figure 10d,d_2_). In the sediment of the SSHC_180_ sample with PR, the contribution of Mg decreased by 5 times, the contribution of Ca by 1.6 times, and the contribution of K by 1.4 times (Figure 10(d_1_,d_3_)).

In the images of the SIHC samples, there is a visible similar tendency of changes in concentration of inorganics: compared to the SIHC_150_ sample, the contribution of Ca increased from 9.09 to 11.76 wt% in the inner part of the SIHC_150_ sample pyrolyzed with PR, and the contribution of Mg increased from 0.43 to 0.47% wt%, but the contribution of K decreased almost twofold (Figure 11c,c_2_). In the sediments of these samples, the changes were as follows: the contribution of Mg increased from 1.25 to 1.29 wt%, and the contribution of K increased from 11.53 to 26.36 wt%, but the contribution of Ca decreased from 46.26 to 34.93 wt% (Figure 11(c_1_,c_3_)). Compared to the corresponding pyrolyzed sample in the composition of the inner part of the SIHC_180_ sample with PR, the contribution of K increased substantially, whereas the contributions of Mg (from 0.24 to 0.15 wt%) and Ca (from 10.69 to 5.74 wt%) decreased (Figure 11d,d_2_). In the sediment on the surface of tablets of the SIHC_180_ sample pyrolyzed with PR, the contribution of K increased by almost 23 times, while the contribution of Ca decreased by almost 1.9 times. The contribution of Mg decreased from 1.05 to 0.88 wt% (Figure 11(d_1_,d_3_)).

The data presented above allow for a preliminary suggestion about the tendencies of changes taking place in the composition of sediments of SSHCs and SIHCs. The addition of PR during pyrolysis of the tablets inhibits the migration of Mg and Ca with volatile products and their deposition in the form of sediment, while the migration of K increases. The observed changes in the contribution of inorganics in volatile products may affect their composition by catalyzing secondary reactions that reduce the emission of undesirable compounds. Therefore, there are certain grounds to suggest that the presence of K migrating with volatiles during the pyrolysis of HCs with PR may have a positive effect on the modification of the composition of the emitted volatile products.

## 3. Materials and Methods

### 3.1. Materials

The biomass under study was obtained directly from farmland. Upon delivery to the laboratory, the samples of sunflower stems (SSs) and sunflower inflorescences (SIs) originating from the same plant were initially ground and washed with distilled water. After drying at room temperature, the biomass was dried at the temperature of 105 °C. Next, the plant material was ground to an average particle size of less than 0.2 mm. The biomass prepared this way was subjected to hydrothermal treatment. The materials of raw biomass, solid residues (hydrochars), and liquid products (aqueous hydroliquors) dried to a constant weight after hydrothermal treatment were densified. To modify the composition of the volatile pyrolysis products of hydrochar, the expired APAP tablets manufactured by US Pharmacia Sp. z o.o. (Ziebicka Str. 40, Wroclaw, Poland) were used. The pharmacologically active substance of this medicine is paracetamol (called N-(4-hydroxyphenyl) acetamide according to the IUPAC nomenclature). Previous studies on how paracetamol influences the transformations taking place in carbon materials have shown that the products of its decomposition can affect the disproportionation of hydrogen during thermochemical transformations and the yield of pyrolysis products [104]. Based on the principles of circular economy with respect to better economization of the process, the active substance was not purified. The paracetamol (PR) tablets were crushed to a grain size of <0.1 mm and added to dried hydrochars in the amount of 2 wt%. The average densities of the obtained tablets are presented in Table 3.

### 3.2. Methods

**Figure 14:**
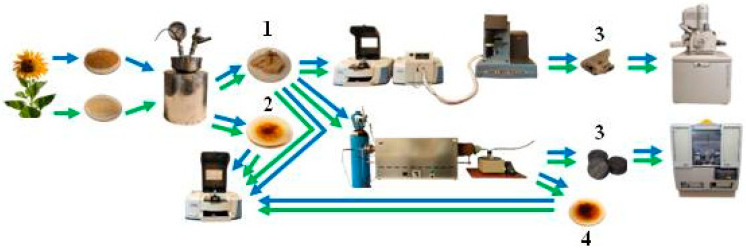
Scheme of the experiment (1—HC, 2—AHL, 3—pyrolyzed HC, and 4—condensate).

Figure 14 presents a schematic diagram of the experiment aimed to conduct the autohydrolysis of SS and SI samples and to investigate the characteristics of the obtained products of this process.

The ground samples were pretreated hydrothermally; the obtained suspension was separated; and then, the hydrochars (HCs) and aqueous hydroliquors (AHLs) were dried to constant weight. Next, their structural–chemical parameters were determined using the ATR technique. The SS, SI, and HC samples were pyrolyzed in a TG/FTIR unit, a Q50 thermobalance manufactured by TA Instruments Inc., 159 Lukens Drive, New Castle, DE, USA coupled with a Nicolet iS10 spectrometer manufactured by Thermo Fisher Scientific Inc., 168 Third Avenue, Waltham, MA, USA, in order to determine the composition of volatile pyrolysis products. The obtained pyrolyzed hydrochars were visualized using a scanning electron microscope (SEM) manufactured by FEI Company, 5350 NE Dawson Creek Drive, Hillsboro, OR, USA, and an EDS microanalysis was carried out. Additionally, the SS, SI, and HC samples were pyrolyzed in a tube furnace. The samples obtained this way were examined by the XRD technique (PANalytical Ltd., Enigma Business Park, Grovewood Road, Malvern, UK), and the material-condensed methanol by the ATR technique.

#### 3.2.1. Hydrothermal Treatment of Samples with Hot Liquid Water (Autohydrolysis)

The autohydrolysis was conducted in a Miniclave steel type 3 manufactured by Büchi AG (Meierseggstrasse 40, Flawil, Switzerland). Amounts of 5 g of raw material and 150 mL of redistilled water were introduced into a 300 mL autoclave. The autoclave was filled with high-purity nitrogen, sealed, and placed in a tube furnace of 800 W manufactured by Termtech Company (ul. Marywilska 39, Warsaw, Poland) that was set on a Multi-position magnetic stirrer SB161-3 (Cole-Parmer Instrument Company, 5 East Bunker Court, Vernon Hills, IL USA). Autohydrolysis is a technological process carried out in deionized liquid water under pressure. It is widely accepted that the temperature range for autohydrolysis is limited by the upper value of approximately 200 °C. Therefore, in this research, three middle temperatures were selected from the range of 100–200 °C, which differ from each other by 30 °C. The autohydrolysis was carried out under the pressure of 2.5–10 bar at three temperatures—120 °C, 150 °C, and 180 °C. The applied temperatures were similar to those used by other researchers [43,105,106]. The samples were kept isothermally under these conditions for 30 min. Then, the autoclave was cooled to ambient temperature, and next, the suspension of hydrochar (HC) and aqueous hydroliquor (AHL) was separated on a Buchner funnel and subjected to further steps. The HCs were dried to constant weight in a VDL23 vacuum drier manufactured by Binder GmbH (Im Mittleren Ösch 5, Tuttlingen, Germany). After, water was distilled from the AHLs; they were dried to constant weight.

#### 3.2.2. Elemental Analysis and Determination of Ash Content

The elemental analysis was conducted using an Elementar Vario Micro Cube CHNS analyzer (Elementar Analysensysteme GmbH, Elementar-Straße 1, Langenselbold, Germany). The determination of ash was carried out according to the Standard Test Method for Ash in Biomass ASTM E1755-01 (2015) [107]. The obtained values of the amount of C, H, N, and S elements as well as the measured amount of ash were used for the determination of the content of O^diff^ element by the differential method (ASTM E870) [108] and for the calculation of the HHV parameter according to the formula proposed by Channiwala and Parikh [109]. A Thermo Scientific Niton Goldd + (Thermo Fisher Scientific Inc.) analyzer was used for the determination of inorganic elements of the selected samples. The following reference materials were used to determine the concentration of elements: NIST-1575a (pine needles), NIST-1573a (tomato leaves), and IC-INCT-PVLT-6 (tobacco leaves).

#### 3.2.3. Pyrolysis in a TG/FT-IR Analytical Unit

The pyrolysis of densified biomass samples, HCs, and blends of HCs with PR was carried out in a Q50 thermobalance manufactured by Thermo Analytical (TA Instruments Inc.) in a high-purity nitrogen flow. The nitrogen flow through the thermobalance was 10 mL·min^−1^, and through the interface 90 mL·min^−1^. The samples were heated to the temperature of 750 °C with a heating rate of 10 °C·min^−1^. The decomposition products were directed to the interface via a transfer line, while the FT-IR spectra of the volatile products were registered by a Nicolet iS10 spectrometer manufactured by Thermo Fisher Scientific Inc. The graphs of the dependence of weight loss (TGA) and weight loss rate on the temperature (DTG) were registered during pyrolysis. The DTG curves of raw biomass were deconvoluted according to the methodology presented in [82,110]. The deconvolution method allows for determination of the contribution of biomass components, the thermal degradation of which causes the formation of the DTG curve. For this purpose, OMNIC 9.2.86 software (Thermo Fisher Scientific Inc.) was used due to the coupling of Q50 thermobalance with a Nicolet iS10 spectrometer. OMNIC 9.2.86 software was used for the elaboration of FT-IR spectra: corrections of the baseline were performed, and the surfaces of particular bands were determined. The spectra were normalized with respect to the height of the CO_2_ band near 2300 cm^−1^. This operation allowed for a semi-quantitative evaluation of changes in the contribution of particular functional groups and atomic groups in the formation of the FT-IR spectra.

#### 3.2.4. Pyrolysis of Samples in a Pyrolytic Furnace

The densified samples of raw biomass and its HCs were pyrolyzed in a tube furnace (PRC 70 × 708/110 M) manufactured by Czylok company (Czylok Company PRC 70 × 708/110 M, Pszczyńska 336, Jastrzębie-Zdrój, Poland) under high-purity nitrogen atmosphere. The final pyrolysis temperature was 450 °C. The heating rate was identical to that obtained during the pyrolysis in the TG/FT-IR unit and was 10 °C·min^−1^. The volatile products of pyrolysis were directed from the furnace to a methanol scrubber placed in an ice bath. A part of the volatile products was condensed in cooled methanol. After the experiment was completed and methanol was separated, the condensed products were subjected to further spectroscopic analysis.

#### 3.2.5. Obtaining ATR Spectra of Dried Samples of HCs, AHLs, and Condensates

The ATR spectra of HCs, AHLs, and condensed materials were obtained using a Thermo Fisher Scientific Nicolet iS10 spectrometer with a SMART MIRACLE module equipped with a ZnSe monocrystal. Before the spectra were registered, the background was measured to eliminate the moisture and CO_2_ present in the environment. The spectra were registered in the range of 4000–600 cm^−1^. A total of 64 scans were used for the registration. OMNIC9.2.86 software was used to elaborate the spectra, which made it possible to eliminate the non-specific background and to take into account the local optical minima, as well as to perform the deconvolution of fingerprint ranges of the ATR spectra of AHL samples [111]. The reference height was the height of the band located near 1600 cm^−1^, which indicated the presence of C=C bonds. Adjusting the height of this band for all spectra allowed us to present a visual comparison of the shape and height of bands in all ATR spectra.

#### 3.2.6. Obtaining Diffractograms and SEM Images

The HCs pyrolyzed to the temperature of 450 °C were studied using an X’Pert Pro X-ray diffractometer (PANalytical Company). The operating parameters of the diffractometer were as follows: voltage U = 40 kV, amperage I = 45 mA, range 2θ = 10–45 deg., and measurement step = 0.033 deg. The internal standard method was applied. For this purpose, the studied material was ground in an agate mortar. Then, 10% of the internal standard (NaF) was added to each prepared sample, and next, they were mixed thoroughly. Weighing was performed on a Precisa 92SM-202A analytical balance (Precisa Instruments AG, Moosmattstrasse 32, Dietikon, Switzerland) with the accuracy to four decimal places. The illustrative normalization of diffractograms was performed with respect to the height of the (002) band derived from the standard. The analysis of the evolution of the HC structure caused by the increase in temperatures of autohydrolysis and pyrolysis was carried out quantitatively. For this purpose, the ratios of integral intensities of the (200), (1$\bar{1}$0), and (110) reflexes from hydrochars (assigned according to the PDF card of cellulose I*β* 00-056-1718) and the (002) reflex from the pyrolyzates to the (002) reflex from the internal standard NaF were calculated according to the methodology presented in [73].

The samples of tablets of pyrolyzed hydrochars obtained in the thermobalance at the temperature of 750 °C were cut and stuck to a carbon tape placed on the Holter in such a way that made the visualization of the surface and inside of the tablet possible. The visualization was carried out with the use of a scanning electron microscope Quanta 3D FEG manufactured by FEI Company. The SEM images and the results of the EDS microanalysis were obtained with an accelerating voltage of 10 keV under the magnification of M2k.

## 4. Conclusions

The products of the autohydrolysis of sunflower stems (SSs) and sunflower inflorescence (SI) which originated from the same plant were studied. The samples differed from each other in the parameters of their elemental analysis, the contribution of non-structural components, and the content of ash. The autohydrolysis was carried out in an autoclave at temperatures of 120, 150, and 180 °C under pressure. The course of autohydrolysis was characterized by distinct rates of removal of inorganics from the plant material.

The removal of organic and inorganic components from the studied samples during autohydrolysis caused the evolution of the structural–chemical parameters of the obtained hydrochars. The rise in autohydrolysis temperature increased the contribution of polar compounds able to form H-bonds and the contribution of bonds originating from cellulose and lignin in all hydrochars. The autohydrolysis temperature did not affect the contribution of polar compounds in the aqueous hydroliquors of the SS sample but decreased the contribution of these compounds in the aqueous hydroliquors of the SI sample. The rise in autohydrolysis temperature caused a greater ordering of the structure of hydrochars from the SS sample but facilitated a greater ordering of pyrolyzed hydrochars from the SI sample.

The increase in yield of volatile products during pyrolysis in all hydrochars with the rise in autohydrolysis temperature was accompanied by the growth in the amount of undesirable compounds in volatiles emitted to the environment. In the composition of the condensed material obtained during the pyrolysis of hydrochars of the SI sample, a decrease in the contribution of polar compounds and compounds containing a C–O–C bond was observed, while the reverse situation took place in the composition of material condensed during the pyrolysis of hydrochars of the SS sample.

The results of the observed changes in structural–chemical evolutions in the products of autohydrolysis of the studied samples and their pyrolyzates imply the lack of dependency of these changes on the amount of inorganics removed from hydrochars. The lack of proportionality between the amount of inorganics removed from the hydrochars and the changes occurring in them compels an investigation into the decisive influence of the removal of non-structural components (extractives, pectin, and hemicellulose) from plant material during autohydrolysis.

The non-structural components primarily contribute to the formation of volatile pyrolysis products, which act as carriers for migrating AAEMs. The data from the EDS microanalysis of the sediments imply that, during pyrolysis of HCs with a rise in autohydrolysis temperature, the migration of K disappears, while the migration of Mg and Ca with volatile pyrolysis products increases. The addition of PR to HCs during pyrolysis facilitates the migration of K with volatile products and inhibits the migration of Mg and Ca, which has a positive effect on the changes in the composition of volatile pyrolysis products and on the reduction in pollution of the environment with undesirable compounds.

The use of expired paracetamol as an additive to HCs during pyrolysis can be considered a positive aspect of the transition to the principles of circular economy, which will not only positively modify the composition of volatile pyrolysis products but also allow for a reduction in the costs of disposal of these environmentally hazardous wastes.

## Figures and Tables

**Table 1:** The yields of products of autohydrolysis [%].

Temperature [°C]	SS			SI		
	HC	AHL	Gas	HC	AHL	Gas
120	85	12	3	48	48	4
150	83	14	3	42	43	15
180	72	24	4	37	47	16

**Table 2:** The main characteristics of the raw samples and their hydrochars.

Parameter	C^d^ [%]	H^d^ [%]	N^d^ [%]	S^d^ [%]	O^diff^ [%]	A^d^ [%]	HHV [MJ·kg^−1^]
SS raw	42.66 ± 0.10	5.42 ± 0.10	0.25 ± 0.07	0.10 ± 0.09	51.57 ± 0.50	7.34 ± 0.14	15.90 ± 0.14
SSHC_120_	44.84 ± 0.02	5.77 ± 0.11	0.18 ± 0.07	0.10 ± 0.09	49.11 ± 0.42	2.81 ± 0.13	17.41 ± 0.44
SSHC_150_	45.34 ± 0.10	5.75 ± 0.07	0.14 ± 0.01	0.03 ± 0.10	48.74 ± 0.41	2.24 ± 0.13	17.54 ± 0.11
SSHC_180_	45.82 ± 0.02	5.81 ± 0.02	0.14 ± 0.01	0.01 ± 0.01	48.22 ± 0.15	1.39 ± 0.09	17.83 ± 0.13
SI raw	38.98 ± 0.11	5.25 ± 0.02	1.25 ± 0.05	0.09 ± 0.09	54.43 ± 0.42	9.71 ± 0.15	14.30 ± 0.06
SIHC_120_	42.66 ± 0.26	5.82 ± 0.18	1.48 ± 0.24	1.48 ± 0.24	49.99 ± 0.87	5.48 ± 0.12	16.44 ± 0.29
SIHC_150_	45.44 ± 0.24	5.76 ± 0.14	1.52 ± 0.07	0.09 ± 0.05	47.19 ± 0.63	5.34 ± 0.13	17.65 ± 0.22
SIHC_180_	47.58 ± 0.11	5.82 ± 0.07	1.64 ± 0.06	0.04 ± 0.01	44.92 ± 0.36	4.36 ± 0.11	18.72 ± 0.08

^d^—dry basis; calculation of O^dif^ = 100 − C^d^ − H^d^ − N^d^ − S^d^ − A^d^; calculation of HHV [MJ·kg^−1^] = 0.3491·C^d^ + 1.1783·H^d^ + 0.1005·S^d^ − 0.0151·N^d^ − 0.1034·O^a^ − 0.0211·A^d^.

**Table 3:** Densities of the tablets.

Samples	Density [g·cm^−3^]	
	SS	SI
raw	1.13 ± 0.08	1.10 ± 0.09
HC_120_	1.19 ± 0.03	1.23 ± 0.06
HC_150_	1.22 ± 0.17	1.30 ± 0.03
HC_180_	1.28 ± 0.12	1.32 ± 0.06
HC_150_ with PR	1.23 ± 0.06	1.31 ± 0.07
HC_180_ with PR	1.27 ± 0/09	1.31 ± 0.08

## Data Availability

The data presented in this study are available from the corresponding author on request.

## Supplementary Materials

The following supporting information can be downloaded at https://www.mdpi.com/article/10.3390/molecules31081236/s1, Table S1: The content of selected inorganic elements in the studied samples [%]; Table S2: The calculations of the sub-peak surfaces for the selected fingerprint area; Table S3: The ratio of integral intensity of selected reflexes to the integral intensity of (002) reflex from NaF; Table S4: Surfaces of the selected bands in FT-IR spectra and ratios of the surfaces of these bands to the surface of the CO_2_ band; Table S5: Increase in the surface of the selected bands in FT-IR spectra of volatile products during the pyrolysis of hydrochars; Table S6: Surfaces of selected bands in FT-IR spectra and ratios of surfaces of these bands to the surface of CO_2_ band; Table S7. Changes in surface of selected bands in FT-IR spectra of volatile products during pyrolysis of hydrochars without and with PR.

## Abbreviations

The following abbreviations are used in this manuscript:

AAEMs	alkali and alkaline earth metals
SS	sunflower stem
SI	sunflower inflorescence
HHV	higher heating value
HC	hydrochar
AHL	aqueous hydroliquor
SSHC	hydrochar of SS sample
SIHC	hydrochar of SI sample
SSAHL	aqueous hydroliquor of SS sample
SIAHL	aqueous hydroliquor of SI sample
$A_{\left(1\bar{1}0\right)+\left(110\right)}$	integral intensity of (1$\bar{1}0$) and (110) reflexes
A_NaF_	integral intensity reflex from NaF
