# Supplementary material for: The Influence of Autohydrolysis Temperature and the Addition of 2 wt% of Expired Paracetamol on the Thermal Behavior and Composition of Pyrolysis Products After Hydrothermal Treatment of Sunflower Stems (SSs) and Sunflower Inflorescences (SIs)
Source: Molecules. 2026 Apr 9;31(8):1236. doi: 10.3390/molecules31081236 (PMC13118340; doi:10.3390/molecules31081236)
Supplement: Supplementary file 1 [file molecules-31-01236-s001.zip › Table S1.pdf]

Table S1. The content of selected inorganic elements in studied samples [%]

Sample	Al	Si	P	S	Cl	K	Ca
SS raw	2.26±0.51	8.39±0.37	3.06±0.17	1.97±0.10	5.70±0.07	61.98±0.53	16.06±0.468
$SSHC_{120}$	1.79±0.28	6.32±0.23	0.74±0.08	1.08±0.06	0.64±0.02	12.87±0.21	12.81±0.31
$SSHC_{150}$	1.11±0.20	4.93±0.18	0.34±0.06	0.82±0.10	0.29±0.07	5.42±0.13	10.27±0.25
$SSHC_{180}$	1.03±0.18	4.27±0.16	0.20±0.05	0.66±0.06	0.23±0.02	3.47±0.10	7.53±0.21
