# Supplementary material for: The Influence of Autohydrolysis Temperature and the Addition of 2 wt% of Expired Paracetamol on the Thermal Behavior and Composition of Pyrolysis Products After Hydrothermal Treatment of Sunflower Stems (SSs) and Sunflower Inflorescences (SIs)
Source: Molecules. 2026 Apr 9;31(8):1236. doi: 10.3390/molecules31081236 (PMC13118340; doi:10.3390/molecules31081236)
Supplement: Supplementary file 1 [file molecules-31-01236-s001.zip › Table S2.pdf]

Table S2. The calculations of the sub-peak surfaces for the selected fingerprint area

Samples	$SSAHL_{120}$	$SSAHL_{150}$	$SSAHL_{180}$	$SIAHL_{120}$	$SIAHL_{150}$	$SIAHL_{180}$
substituted alkenes	21.9	23.5	23.6	10.5	-	-
anhydrides	12.7	9.4	10.5	-	29.3	-
alcohols	13.0	13.1	13.3	19.8	23.6	54.1
phenol	4.9	5.5	5.6	8.0	10.1	7.3
ethers	13.4	12.5	12.5	22.5	1.2	1.3
alkanes	3.5	3.6	3.6	2.4	-	-
acids	4.7	5.7	5.6	-	-	-
aromatics	4.3	6.8	6.8	19.2	21.5	21.4
aldehydes, ketones, esters, laktones	19.4	20	18.4	17.4	13.5	15.6
