# Supplementary material for: The Influence of Autohydrolysis Temperature and the Addition of 2 wt% of Expired Paracetamol on the Thermal Behavior and Composition of Pyrolysis Products After Hydrothermal Treatment of Sunflower Stems (SSs) and Sunflower Inflorescences (SIs)
Source: Molecules. 2026 Apr 9;31(8):1236. doi: 10.3390/molecules31081236 (PMC13118340; doi:10.3390/molecules31081236)
Supplement: Supplementary file 1 [file molecules-31-01236-s001.zip › Table S3.pdf]

Table S3. The ratio of integral intensity of selected reflexes to the integral intensity of (002) reflex from NaF.

Samples	Hydrochar		Pyrolysate
	$A_{(1\bar{1}0)+(110)}/A_{(002)NaF}$	$A_{(200)}/A_{(002)NaF}$	$A_{(002)}/A_{(002)NaF}$
SS raw	1.05	3.57	0.83
SSHC 120	1.78	5.36	1.89
SSHC 150	1.86	5.60	2.26
SSHC 180	2.27	5.63	2.39
SI raw	0.48	1.72	1.32
SIHC 120	0.89	2.98	2.25
SIHC 150	0.94	3.13	2.41
SIHC 180	1.25	3.97	2.73
