# Supplementary material for: The Influence of Autohydrolysis Temperature and the Addition of 2 wt% of Expired Paracetamol on the Thermal Behavior and Composition of Pyrolysis Products After Hydrothermal Treatment of Sunflower Stems (SSs) and Sunflower Inflorescences (SIs)
Source: Molecules. 2026 Apr 9;31(8):1236. doi: 10.3390/molecules31081236 (PMC13118340; doi:10.3390/molecules31081236)
Supplement: Supplementary file 1 [file molecules-31-01236-s001.zip › Table S4.pdf]

Table S4. Surfaces of selected bands in FT-IR spectra and ratios of surfaces of these bands to the surface of CO_2 band.

Sample	PAHs $A_{3200-2600}$	CO_2 $A_{2400-2230}$	C=O $A_{1900-1600}$	Phenols $A_{1255-1135}$	Alcohols $A_{1135-1050}$	A_{PAHS}/ A_{CO2}	$A_{C=O}$/ A_{CO2}	$A_{phenokls}$/ A_{CO2}	$A_{alkohols}$/ A_{CO2}
SS raw	6702	26457	18455	5780	7400	0.253	0.698	0.218	0.280
$SSHC_{120}$	8051	18722	18137	6377	6533	0.430	0.969	0.341	0.349
$SSHC_{150}$	8062	18214	16265	5831	5774	0.443	0.893	0.320	0.282
$SSHC_{180}$	6805	14462	12509	4463	4078	0.471	0.865	0.309	0.317
SI raw	2527	22038	8742	2242	3502	0.115	0.397	0.102	0.158
$SIHC_{120}$	3781	16954	9372	2576	3369	0.223	0.553	0.152	0.199
$SIHC_{150}$	4672	19056	12313	3555	4802	0.245	0.646	0.1865	0.252
$SIHC_{180}$	3716	11781	8683	2223	3091	0.315	0.737	0.189	0.262
