# Supplementary material for: The Influence of Autohydrolysis Temperature and the Addition of 2 wt% of Expired Paracetamol on the Thermal Behavior and Composition of Pyrolysis Products After Hydrothermal Treatment of Sunflower Stems (SSs) and Sunflower Inflorescences (SIs)
Source: Molecules. 2026 Apr 9;31(8):1236. doi: 10.3390/molecules31081236 (PMC13118340; doi:10.3390/molecules31081236)
Supplement: Supplementary file 1 [file molecules-31-01236-s001.zip › Table S5.pdf]

Table S5. Increase in surface of selected bands in FT-IR spectra of volatile products during pyrolysis of hydrochars

Band ratios	3200-2600 cm^{-1}	1900-1600 cm^{-1}	1255-1135 cm^{-1}	1135-1050 cm^{-1}
$SSHC_{120}$/SS raw	1.70	1.39	1.56	1.25
$SSHC_{150}$/SS raw	1.74	1.28	1.47	1.13
$SSHC_{180}$/SS raw	1.86	1.24	1.42	1.04
$SIHC_{120}$/SI raw	1.94	1.39	1.49	1.25
$SIHC_{150}$/SI raw	2.13	1.63	1.83	1.58
$SIHC_{180}$/SI raw	2.74	1.85	1.85	1.65
