# Supplementary material for: The Influence of Autohydrolysis Temperature and the Addition of 2 wt% of Expired Paracetamol on the Thermal Behavior and Composition of Pyrolysis Products After Hydrothermal Treatment of Sunflower Stems (SSs) and Sunflower Inflorescences (SIs)
Source: Molecules. 2026 Apr 9;31(8):1236. doi: 10.3390/molecules31081236 (PMC13118340; doi:10.3390/molecules31081236)
Supplement: Supplementary file 1 [file molecules-31-01236-s001.zip › Table S6.pdf]

Table S6. Surfaces of selected bands in FT-IR spectra and ratios of surfaces of these bands to the surface of CO_2 band.

Sample	PAHs $A_{3200-2600}$	CO_2 $A_{2400-2230}$	C=O $A_{1900-1600}$	Phenols $A_{1255-1135}$	Alcohols $A_{1135-1050}$	A_{PAHS}/ A_{CO2}	$A_{C=O}$/ A_{CO2}	$A_{phenokls}$/ A_{CO2}	$A_{alkohols}$/ A_{CO2}
$SSHC_{150}$	8062	18214	16265	5831	5774	0.443	0.893	0.320	0.282
$SSHC_{150}$ with PR	7389	13256	16400	4814	5499	0.557	1.237	0.363	0.415
$SSHC_{180}$	6805	14462	12509	4463	4078	0.471	0.865	0.309	0.317
$SSHC_{180}$ with PR	7589	14476	15527	5314	5106	0.524	1.073	0.367	0.353
$SIHC_{150}$	4672	19056	12313	3555	4802	0.245	0.646	0.1865	0.252
$SIHC_{150}$ with PR	3352	13082	9070	3195	2174	0.256	0.693	0.244	0.240
$SIHC_{180}$	3716	11781	8683	2223	3091	0.315	0.737	0.189	0.262
$SIHC_{180}$ with PR	2983	13974	8627	2872	2168	0.213	0.617	0.206	0.155
