# Supplementary material for: The Influence of Autohydrolysis Temperature and the Addition of 2 wt% of Expired Paracetamol on the Thermal Behavior and Composition of Pyrolysis Products After Hydrothermal Treatment of Sunflower Stems (SSs) and Sunflower Inflorescences (SIs)
Source: Molecules. 2026 Apr 9;31(8):1236. doi: 10.3390/molecules31081236 (PMC13118340; doi:10.3390/molecules31081236)
Supplement: Supplementary file 1 [file molecules-31-01236-s001.zip › Table S7.pdf]

**Table S7**. Changes in surface of selected bands in FT-IR spectra of volatile products during pyrolysis of hydrochars without and with PR

| Band ratios | 3200-2600 $cm^{-1}$ | 1900-1600 $cm^{-1}$ | 1255-1135 $cm^{-1}$ | 1135-1050 $cm^{-1}$ |
|---|---|---|---|---|
| $SSHC_{150}$/ $SSHC_{150}$ with PR | 0.8 | 0.7 | 0.9 | 0.7 |
| $SSHC_{180}$/ $SSHC_{180}$ with PR | 0.9 | 0.8 | 0.8 | 0.9 |
| $SIHC_{150}$/ $SIHC_{150}$ with PR | 1.0 | 0.9 | 0.8 | 1.1 |
| $SIHC_{180}$/ $SIHC_{180}$ with PR | 1.5 | 1.2 | 1.3 | 1.5 |
